# MP4: a machine learning based classification tool for prediction and functional annotation of pathogenic proteins from metagenomic and genomic datasets

**DOI:** 10.1186/s12859-022-05061-7

**Published:** 2022-11-28

**Authors:** Ankit Gupta, Aditya S. Malwe, Gopal N. Srivastava, Parikshit Thoudam, Keshav Hibare, Vineet K. Sharma

**Affiliations:** MetaBioSys Group, Department of Biological Sciences, Indian Institute of Science Education and Research, Bhopal, Madhya Pradesh India

**Keywords:** Pathogenesis, Pathogenic proteins, Machine learning, SVM

## Abstract

Bacteria can exceptionally evolve and develop pathogenic features making it crucial to determine novel pathogenic proteins for specific therapeutic interventions. Therefore, we have developed a machine-learning tool that predicts and functionally classifies pathogenic proteins into their respective pathogenic classes. Through construction of pathogenic proteins database and optimization of ML algorithms, Support Vector Machine was selected for the model construction. The developed SVM classifier yielded an accuracy of 81.72% on the blind-dataset and classified the proteins into three classes: Non-pathogenic proteins (Class-1), Antibiotic Resistance Proteins and Toxins (Class-2), and Secretory System Associated and capsular proteins (Class-3). The classifier provided an accuracy of 79% on real dataset-1, and 72% on real dataset-2. Based on the probability of prediction, users can estimate the pathogenicity and annotation of proteins under scrutiny. Tool will provide accurate prediction of pathogenic proteins in genomic and metagenomic datasets providing leads for experimental validations. Tool is available at: http://metagenomics.iiserb.ac.in/mp4.

**Supplementary Information:**

The online version contains supplementary material available at 10.1186/s12859-022-05061-7.

## Introduction

Pathogenic microbes possess unique virulent factors encoded by their genes present on chromosomal DNA that may exist as pathogenicity islands, or in their extrachromosomal plasmids [1]. High-throughput genomic analysis has shown that there is a significant difference in the genome of pathogenic and non-pathogenic bacteria even within closely-related species [2]. Such differences have been exploited to develop tools which predict pathogenic genes in genomes and metagenomes [3]. In addition to being species-specific or host-specific, pathogenesis processes are multifaceted broadly consisting of virulence, adhesion, invasion, secretion, and drug resistance [4]. As a result, vast differences exist in the protein profile of pathogenic and non-pathogenic bacteria as pathogenic bacteria require proteins which can aid them in survival and proliferation within the host during infection. For example, pathogenic and non-pathogenic *Listeria* species show significant difference in their proteome [5]. Similarly, pathogenic *Mycobacterium tuberculosis* and non-pathogenic *Mycobacterium smegmatis* show differences in porin complexes in their outer membrane [6]. However, many of the putative outer membrane proteins of *Mycobacterium tuberculosis* have not been yet been identified and characterized [7], many of which may be responsible in its virulence and survival in host cells.

Taken together, the above examples indicate that there is an urgent need to understand the role of unknown or yet unannotated proteins in pathogenesis, which can be considered as leads for further experiments.

Homology-based approaches are able to provide only limited information for identification and functional annotation of virulence determinants in bacteria [8], hence machine learning (ML)-based approaches provide ideal substitutes for predicting and identifying virulence determinants. Given the plethora of biological information available in the form of sequence data, attempts can be made to use this data to predict pathogenic proteins using machine learning and artificial intelligence approaches. The high-quality and manually curated data can be used for training and testing the ML models followed by performance evaluation to develop efficient and reliable classifiers for prediction of pathogenic proteins. Several algorithms like Random forest, SVM, kNN etc. are available that are known to provide excellent performances in such classifiers [3, 9, 10].

Initial studies were focused on the classification of human pathogenic and non-pathogenic bacteria based on presence or absence of pathogenic protein profiles [11, 12]. Another binary classifier called *PaPrBaG* [13] based on Random forest algorithm can identify pathogens from Next Generation Sequencing Data. However, these tools can only differentiate between bacterial human pathogens from non-pathogen. VirulentPred [14], an SVM-based tool, was developed to predict bacterial virulence proteins. This was followed by another tool *MP3* [3], which is one of the most commonly used tool to predict bacterial pathogenic proteins in both genomic and metagenomic datasets using an integrated SVM-HMM approach and provides an accuracy up to 89%. However, both these tools are binary classifiers that predict if a protein will be pathogenic or non-pathogenic and do not provide a functional annotation. In summary, there is a deficit of tools that can identify the role of virulent protein in the multistep process of pathogenesis and can also annotate the novel proteins identified in new sequenced genomes. PathoFact is a recently developed pipeline that identifies antimicrobial resistance genes and bacterial toxins from metagenomic datasets [15]. In this study, we constructed a unique classifier “MP4” that predicts pathogenic proteins in both genomic and metagenomic datasets, and categorizes the protein into the two classes of pathogenesis along with its predicted annotation, which is helpful to understand role of the protein in the process of pathogenesis.

## Materials and methods

### Dataset preparation

The datasets of pathogenic proteins were constructed by curating the available databases such as Virulence Factor Database (VFDB) [16], Pathosystems Resource Integration Center (PATRIC) [17], PHIDIAS virulent factors (http://www.phidias.us/victors/) [18], Antibiotic Resistance Proteins from Comprehensive Antibiotic Resistance Database (CARD) [19] and Antibiotic Resistance Genes Database (ARDB) [20]. The initial dataset contained 8,794 sequences (excluding antibiotic resistance proteins) as virulent proteins and 4,992 Antibiotic Resistance sequences. A negative dataset consisting of non-pathogenic proteins comprised of a total of 18,296 sequences was also constructed using the Database of Essential Genes [21].

The sequences with non-confirmatory annotations such as “hypothetical”, “like”, “may”, “possible”, “potential”, “predicted”, “probable”, “putative”, “uncharacterized”, “unknown”, “unnamed” were removed from the main dataset and were used for constructing the Real Dataset. For removing the redundancy in the remaining sequence data, clustering was performed using CD-HIT at a cut-off value of 0.95 (i.e., 95% sequence similarity) and the representative sequences from each cluster were obtained [22]. The clustering resulted in the sets of 4948 virulent proteins sequences (not including antibiotic resistance proteins), 1040 antibiotic resistance protein sequences and 11,029 non-pathogenic proteins.

#### Sub-categorization of datasets and curation into multiple classes

The sub-categorization was performed based on the function of the protein sequences followed by an extensive literature review to classify the different groups of proteins into broader categories. The uniqueness and the relatedness amongst the different classes of proteins in terms of their role in pathogenicity, structural diversity, and the origin of the proteins, were considered while classifying the proteins. Sequences with ambiguous annotations were removed to reduce the noise in the data. Sequences in the essential proteins database having similar annotations to virulent proteins were removed. Additionally, except polymerases, enzyme sequences were removed to decrease large variability within negative dataset.

Thus, MP4 protein dataset consisted of protein sequences belonging to 6 major classes: Antibiotic resistance proteins, Non-pathogenic proteins, Secretory proteins, Sigma proteins, Capsules and toxins. Non-pathogenic proteins were considered as a single class, the Antibiotic resistance proteins and Toxins were clubbed into a single class because both these types of protein have either evolved or adapted for the sole purpose of bacterial resistance and pathogenicity, while the other class comprising of secretory proteins and capsular proteins aids in the virulence of a microbe ranging from enhancing attachment to the optimization of resources in the bacterial niche to disruption of host cells. Thus, the sequences were classified as: Class 1 proteins consisting of Non-pathogenic proteins (1047 sequences), Class 2 proteins consisting of antibiotic resistance proteins and toxins (1020 sequences), and Class 3 proteins with secretory and capsular proteins (1492 sequences).

Each class was randomly divided into 80:20 ratios from which the 80% parts from all the classes were combined to make a training dataset (2848 sequences). Blind dataset contained rest 20% of the dataset (711 sequences), based on which statistical measures of data classification of machine learning model is estimated.

#### Construction of different validation datasets

For performance validation of MP4, two different datasets were used consisting of sequences not used for training. Sequences kept for the construction of real dataset-1, were curated into the three classes according to the aforementioned procedure. The real dataset-1 consisted of 308 manually curated sequences. A real dataset-2 consisting of 200 sequences previously used in MP3 [3], was also curated based on the sub-categorization mentioned in the previous section.

#### Construction of independent genomic and metagenomic validation datasets and comparative datasets

A literature review was performed to construct independent datasets consisting of 25 pathogenic and 25 non-pathogenic bacterial strains, respectively. These sequences were then downloaded from the NCBI FTP server (ftp://ftp.ncbi.nlm.nih.gov/).

The performance comparison between MP3 and MP4 was performed using the VirulentPred sequences [14] and *Shigella flexineri* virulence plasmid sequences which consisted of *Shigella flexineri* virulence plasmid group I and *Shigella flexineri* virulence plasmid group II. *Shigella flexineri* virulence plasmid group I consists of proteins that are translocated by *Shigella* into the host cells during the infection (Translocated proteins, 18 sequences) whereas *Shigella flexineri* virulence plasmid group II contains proteins that remain in the bacteria during the infection (Non-translocated proteins, 19 sequences), which were previously used for the validation of the performance of MP3 [3]. The *Shigella flexineri* virulence plasmid group III (1 sequence) [3] was also used to validate the function of MP4. All the sequences used to compare MP3 and MP4 were previously used by MP3 [3]. A third dataset consisting of 41 proteins from *Mycobacterium tuberculosis* NITR203 strain that included known as well as hypothetical proteins was used to assess ability of MP4 to predict and sub-categorize hypothetical sequences. These sequences were earlier used by MP3 [3].

Metagenomic datasets for healthy individual (SRR5898979) and colorectal cancer patient (SRR8865601) were obtained from [23]. Using SPAdes (version 3.13.0) [24], forward and reverse pairedend reads were assembled into single reads for CRC patient and healthy individual respectively. Using Prodigal (version 2.6.3) software [25], gene prediction was performed which were used as an input for MP4 to predict proportion of different classes of pathogenic proteins in the two samples.

##### Calculation of pathogenicity index

The pathogenicity index was calculated for all the strains and was used as basis for identifying and differentiating pathogenic strains from non-pathogenic strains.

$$Pathogenicity\,Index=\frac{Number\,of\,positive\,sequences}{Total\,number\,of\,sequences}$$

where;

$$Number\,of\,positive\,sequences = Number\,of\,sequences \in class 2+Number\,of\,sequences\in Class 3$$

### Input features

#### Dipeptide frequency and pepstats features

The dipeptide frequency provides information about the amino acid sequence arrangement for a protein. As an input, it provides global information on the protein features in a fixed-length vector. It encompasses information such as local order and fraction of amino acid. The dipeptide frequency of each protein can be calculated using the following formula:

$$Dipepide\,frequency=\frac{Total\,number\,of\,dipeptides}{Total\,number\,of\,all\,possible\,dipeptides}\times 100$$

The pepstats features were calculated using EMBOSS:6.6.0.0 (http://emboss.sourceforge.net/apps/cvs/emboss/apps/embossupdate.html). The pepstats calculation provided a total of 34 features consisting of physicochemical properties of proteins including Molecular weight, Number of residues, Average residue weight, Charge, Isoelectric point, molar percent and extinction coefficient at 1 mg/ml (A280) (Additional file 1: Table S1). With these features, the sequences with variable lengths were converted into vectors with Lx434 dimension (L: number of rows in each dataset), which our machine learning algorithm can use for data classification.

### Selection of appropriate machine learning model for classification

WEKA 3.8.2 was used to compare the various machine learning algorithms including PART, Random Forest, IBk and SVM using fivefold cross validation on training data with dipeptide frequency and pepstats features as inputs. Accuracy, precision, F-measure, MCC, ROC, true positive rate (TPR) and true negative rate (TNR) values were recorded for all the algorithms mentioned in this section.

### Optimization of various RF and SVM parameters for the development of classification models

The comparison between different machine learning algorithms revealed that SVM and RF had comparable performances in terms of Accuracy, Precision, F-measure, MCC and ROC values on the training dataset. Hence, both SVM and RF were considered for further parameter optimization and evaluation using e1071 package [26] and randomForest library respectively, available in R (version 3.4). For the RF-based model, the importance of each feature was calculated at *ntree* = 500, using the mean decrease in accuracy at best mtry obtained with the help of *tuneRF* function which calculates *mtry* values using OOB error as an estimate. The OOB error that represents the error in prediction by randomForest algorithm was calculated using top 5, 10, 15, 20, 30, 50, 70 and 90% features (dipeptide frequencies + pepstats features) using different *mtry* values at *ntree* = 200–1000. The best performing model was selected based on the least %OOB error. The performance of various classification models employing RF algorithms was optimized using fivefold cross-validation. In RF, the OOB error was used as a measure for accuracy.

The tuning of SVM kernels was performed using *tune* function of R library e1071 at different values of cost, gamma, degree and coef0 with the help of all 434 features (Additional file 2: Table S2). Parameters for the kernel having the highest total cross-validation accuracy and the least error were selected for the development of SVM-based models. The SVM-based feature selection was performed using the *VarImp* function, a generic method for calculating variable importance for objects. The *VarImp* function uses ROC values as the measure of the importance of features. Three different lists consisting of important features for each class were obtained. Further, top 50, 100, 150, 200, 250, 300, 350, 400 and 410 features were extracted from each list. Then, for every list, the entries with the same ROC value between all the three classes were extracted to give final lists of top features.

### Performance validation of final classification model

The performances of the final models were evaluated using 20% of total data kept as blind dataset and on the independent datasets consisting of 25 known pathogenic and non-pathogenic strains. Performances of the model are represented in terms of Sensitivity, Specificity, Accuracy, Precision and Balanced Accuracy.

$$Sensitivity\; = \;\frac{TP}{{TP + FN}},\;Specificity\; = \;\frac{TN}{{TN + FN}},\;Precision = \;\frac{TP}{{TP + FP}}$$

$$Accuracy\; = \;\frac{TP + TN}{{TP + FN + FP + TN}}$$

$$Balanced\,Accuracy=\frac{Sn+Sp}{2}$$

$$MCC=\frac{\left(TP*TN\right)-\left(FP*FN\right)}{\surd \left(TP+FP\right)\left(TP+FN\right)\left(TN+FP\right)\left(TN+FN\right)}$$

*Sensitivity (Sn)* Sensitivity measures the ability of the process to predict correct results.

*Specificity (Sp)* Specificity measures the ability of a process to predict incorrect results.

*Accuracy* Accuracy measures the degree of correctness of the predicted results to its actual value or the experimental value.

*MCC* Matthews correlation coefficient.

*Balanced Accuracy* Balanced Accuracy measures the average of the proportion corrects of each class individually. It is used when the dataset used for training purposes is unbalanced.

Where, TP: The pathogenic protein correctly identified as pathogenic.

FP: The non-pathogenic protein incorrectly identified as pathogenic protein.

TN: The non-pathogenic protein correctly identified as non-pathogenic.

FN: The pathogenic protein incorrectly identified as non-pathogenic protein.

## Results

### Selection of machine learning algorithms and optimization parameters

On comparing the various machine learning algorithms, it was observed that both Random Forest (RF) and Support vector machines (SVM) gave comparable results with accuracy, MCC, and ROC values of 74.9%, 0.56, 0.87 and 73.6%, 0.57 and 0.81, respectively (Table 1). Hence, both the algorithms were selected for further parameter optimization.

**Table 1:** Comparison of performances between the algorithms

Values	PART	RF	IBk	SVM
Accuracy (%)	62.9	74.9	66.4	73.6
Precision	0.64	0.69	0.73	0.73
F-Measure	0.64	0.76	0.64	0.75
MCC	0.38	0.56	0.44	0.57
ROC	0.69	0.87	0.71	0.81
TPR	0.63	0.75	0.66	0.74
TNR	0.80	0.85	0.83	0.85

*MCC* Matthews correlation coefficient, *TPR* True positive rate, *TNR* True negative rate

The mean decrease in accuracy of the top 30 features is represented in Additional file 3: Fig. S1 and the complete list of variables with the mean decrease in accuracy values is given in Additional file 1: Table S1. The performances of RF-based classification models were calculated at various *mtry* values and at *ntree* = 200–1000 using different top variables. From Additional file 4: Table S3, Additional file 5: Fig. S2a and Additional file 6: Fig. S2b, it is apparent that the model developed using top 50% features at *mtry* = 13 and *ntree* = 800 performed better than the other RF-based models and displayed the least %OOB error of 22.12%.

Best parameters for different kernels were achieved using cross-validation accuracies calculated at the tuning step of SVM algorithm. Three SVM kernels, linear, polynomial and radial basis function were used for the model development. Of these kernels, the polynomial kernel showed the best performance with the percentage accuracy and error of 80.2% and 0.18, respectively. The results for all the kernels and their parameters are mentioned in Table 2. As mentioned in the materials and method section, the features with the same ROC values calculated by *VarImp* function were extracted for the three classes to generate different lists of top variables consisting of 33, 68, 105, 152, 210, 258, 306, 386, 401 features, respectively. The list of top 386 features selected using *VarImp* function is given in Additional file 7: Supplementary Text. For the selection and development of the final SVM-based classification model, the five-fold cross-validation was performed using the top features and their cross-validation accuracy was recorded. The best performing SVM-based model showed a cross-validation accuracy of 79.88% (Table 3).

**Table 2:** The cross validation performances of all the three kernels at their best parameters

Kernel	Accuracy (%)	Error	Dispersion	Cost	Gamma	Degree	Coefficient
Linear	69.03	0.30	0.01	1	–	–	–
Polynomial	80.2	0.18	0.02	1	0.1	2	2
RBF	78.6	0.2	0.02	6	0.001	–	–

*RBF* Radial basis function

**Table 3:** The cross validation accuracies of SVM-based models at different top features

Top features	Accuracy
33	67.77
68	67.77
105	72.08
152	75.18
210	76.90
387	79.88
401	79.60

Where, Accuracy: Cross validation accuracy in percentage

### Performance evaluation of RF and SVM classification models using blind set

On comparing the performance of RF and SVM based module on the blind dataset, an accuracy of 78.48% and 81.72% was obtained (Additional file 8: Table S4 and Table 4). The class-wise (Class 1, Class 2 and Class 3) sensitivity, specificity and balanced accuracy values of the SVM-based model and RF-based model are mentioned in Table 4 and Additional file 8: Table S4, respectively, which show that the SVM-based model performed better, both at cross-validation step and on the blind dataset. Thus, the SVM-based model with 386 features was considered as the final model, and the performance of this model was validated using real validation dataset (materials and method section). Further evaluation of performance was carried out using independent datasets consisting of bacterial pathogenic and non-pathogenic protein sequences.

**Table 4:** The performance of SVM-based model on the MP4 blind set

Accuracy (%)	81.72		
	Class 1	Class 2	Class 3
Sensitivity	0.81	0.77	0.85
Specificity	0.93	0.93	0.86
Balanced accuracy	0.87	0.85	0.85

Where, Class 1: Non-pathogenic proteins; Class 2: Antibiotic resistance and toxic proteins and Class 3: Secretory and capsular proteins

### Performance validation of MP4 using real datasets

The performance of the SVM-based model was evaluated using the 308 sequences that were curated as real dataset-1 and another set of 200 sequences curated as real dataset-2 (materials and method section) and displayed an accuracy of 79.22% and 72% respectively. The class-wise values of sensitivity, specificity and balanced accuracies are provided in Tables 5 and 6.

**Table 5:** The performance of SVM based model on the real dataset1

Accuracy (%)	79.22		
	Class 1	Class 2	Class 3
Sensitivity	0.88	0.74	0.77
Specificity	0.99	0.89	0.80
Balanced accuracy	0.93	0.82	0.79

Where, Class 1: Non-pathogenic Proteins; Class 2: Antibiotic Resistance and Toxic proteins and Class 3: Secretory and capsular proteins

**Table 6:** The performance of SVM based model on the real dataset2

Accuracy (%)	72.0		
	Class 1	Class 2	Class 3
Sensitivity	0.71	0.56	0.78
Specificity	0.94	0.85	0.80
Balanced accuracy	0.83	0.70	0.79

Where, Class 1: Non-pathogenic Proteins; Class 2: Antibiotic Resistance and Toxic proteins and Class 3: Secretory and capsular proteins

### Performance comparison between MP4, MP3 and VirulentPred using real datasets

#### Using real dataset-1

The performance of MP4 was compared with the previously published and publicly available tools MP3 [3] and VirulentPred [14] on real datasets. On real dataset-1, MP4 was able to predict pathogenicity of many proteins with yet to be confirmed function as compared to MP3 and VirulentPred. For example, MexG protein from *Pseudomonas aeruginosa* was predicted to be non-pathogenic by MP3 and VirulentPred but MP4 predicted it as a class 2 pathogenic protein. The role of MexG in regulation of antibiotic efflux and other virulence factor such as pyocyanin [27] aligns well with the prediction provided by MP4. Similarly, ArlR protein is involved in multifaceted regulation of biofilm formation and pathogenesis in *Staphylococcus aureus* [28], and was predicted to be class 2 pathogenic protein by MP4 whereas MP3 and VirulentPred predicted it to be non-pathogenic. *Vibrio parahaemolyticus* putative protein VPA1351, which hypothesized to be type 3 secretion system apparatus protein [29], was predicted as class 3 pathogenic protein by MP4 as compared to MP3 and VirulentPred that predicted it to be non-pathogenic (Additional file 9: Table S5).

#### Using real dataset-2

In real dataset-2, protein sequences from *Cronobacter turicensis z3032, Erwinia amylovora CFBP1430, Erwinia billingiae Eb661 and Salmonella bongori NCTC12419* showed 100% identity to YchO family inverse autotransporters that belong to Type Ve secretion system [30], and were predicted to be in the class 3 by MP4, which includes secretory type proteins. The *Chromobacterium violaceum ATCC12472* sequence was predicted to belong to class 3 pathogenic proteins. The result was also supported by BLAST result that showed a 100% identity of this protein with the *EscJ/YscJ/HrcJ* family type III secretion inner membrane ring protein found in *Chromobacterium violaceum*. Similarly, *PrgI*, *sipD and sipC* from *Salmonella enterica (serovar typhimurium) LT2* were predicted to belong to the class 3 by MP4 and the results were supported by BLAST which identified them as type III secretion system proteins. *IpaC* from *Shigella flexneri (serotype 2a) 301* was again correctly predicted by MP4, and was predicted to be in class 2 pathogenic proteins, which is supported by the previously published literature where it was shown that *Shigella* exhibited chloroquine resistance [31]. Similarly, Pesticin from *Yersinia pestis CO92* showed 100% identity to TonB-dependent siderophore receptors found in *Enterobacterales* and was correctly predicted to be in class 3 by MP4*.* In another example, MP4 predicted *MdtG* protein of *Klebsiella pneumoniae* subsp. *pneumoniae* MGH78578 to be in class 2. This was confirmed by BLAST analysis which showed 100% identity with *MdtG found in Klebsiella pneumoniae* subsp. *pneumoniae DSM 30104* which is experimentally proven to be involved in the multidrug resistance in *Klebsiella pneumoniae DSM* 30104 [32]. Similarly, tetracycline efflux protein found in *Salmonella enterica* was predicted to be in class 2, which was also supported by the BLAST results. RNA polymerase sigma factors found in *Bacillus anthracis strA0248* were predicted to be in class 2 by MP4 which shows that RNA polymerase sigma factors are essential for antibiotic resistance in *Bacillus anthracis strA0248*. These results were supported by the studies conducted by Ross et al. where they showed that the deletion of sigma factors stops the *β-lactamase* activity associated with *B. Anthracis* [33] (Additional file 10: Table S6).

OrgA subunit involved in secretion of needle subunits of type 3 secretion system in *Burkholderia pseudomallei* [34] was predicted as class3 pathogenic protein by MP4 as compared to non-pathogenic predictions provided by MP3 and VirulentPred. MP3 and VirulentPred also failed to predict pathogenicity of Erm(x) gene product 23S rRNA N-6-methyltransferase [35], which was correctly predicted as class 2 protein predicting its role in antibiotic resistance by MP4. In another such instance, both MP3 and VirulentPred failed to predict pathogenicity of *Yersinia pseudotuberculosis* chaperone protein YscY while MP4 was able to predict as class 3 pathogenic protein, which is supported by earlier study where they showed that YscY chaperone protein is essential prior to formation of type 3 secretion system needle in *Yersinia pseudotuberculosis* [36] (Additional file 10: Table S6).

### Performance validation of MP4 using independent genomic and metagenomic validation datasets

On the independent bacterial pathogenic and non-pathogenic datasets of 25 strains each, a higher number of pathogenic proteins were predicted by MP4 in the pathogenic bacterial protein dataset in comparison to the non-pathogenic bacterial proteins (Tables 7 and 8). In the cases of well-known and properly documented pathogens such as *Bacillus anthracis A2012 uid54101, Chlamydophila pneumoniae TW 183 uid57997, Helicobacter pylori B8 uid49873, Shigella dysenteriae 1617 uid229875, Klebsiella pneumoniae 342 uid59145* and *Salmonella typhimurium DT104 uid223287,* the pathogenicity index values were reported to be 0.86, 0.84, 0.82 0.81, 0.77 and 0.75, respectively. In contrast, in the case of non-pathogenic bacterial genomes such as *Thermotoga maritima MSB8 uid57723, Aquifex aeolicus VF5 uid57765, Mycoplasma hyopneumoniae 7448 uid58039* and *Bacillus coagulans 2 6 uid68053,* the pathogenicity index was calculated and reported to be 0.42, 0.42, 0.56 and 0.57, respectively.

**Table 7:** Performance validation of MP4 on pathogenic protein dataset

Strains	Class 1	Class 2	Class 3	Total sequences	Pathogenicity index	References
Bacillus anthracis A2012 uid54101	42	96	159	297	0.859	https://www.ncbi.nlm.nih.gov/bioproject?cmd=Retrieve&dopt=Overview&list_uids=299
Prevotella melaninogenica ATCC 25,845 uid51377	344	288	1661	2293	0.85	http://hmp.jcvi.org/jumpstart/hmp013/index.shtml
Chlamydophila psittaci 6BC uid63621	154	139	682	975	0.842	https://doi.org/10.1128/mBio.00604-12
Chlamydophila pneumoniae TW 183 uid57997	178	160	775	1113	0.84	PMID: 26420648
Helicobacter pylori B8 uid49873	314	197	1196	1707	0.816	PMID:21896079
Helicobacter pylori SouthAfrica20 uid216150	320	218	1164	1702	0.812	PMID: 21081026
Shigella dysenteriae 1617 uid229875	1224	2520	2665	6409	0.809	
Providencia stuartii MRSN 2154 uid162193	900	920	2279	4099	0.78	
Francisella tularensis holarctica F92 uid181998	407	600	835	1842	0.779	PMC3569339
Escherichia coli CFT073 uid57915	1196	1519	2649	5364	0.777	PMID: 12471157
Proteus mirabilis HI4320 uid61599	817	786	2059	3662	0.777	PMID: 18375554
Klebsiella pneumoniae 342 uid59145	1302	1649	2815	5766	0.774	https://doi.org/10.1371/journal.pgen.1000141
Capnocytophaga ochracea DSM 7271 uid59197	493	616	1062	2171	0.773	PMID: 21304645
Citrobacter koseri ATCC BAA 895 uid58143	1153	1397	2456	5006	0.77	PMID:12751719
Escherichia coli clone D i14 uid162049	1138	1342	2438	4918	0.769	https://doi.org/10.1371/journal.ppat.1006525
Mycoplasma pneumoniae 309 uid85495	164	107	436	707	0.768	PMID:18754792
Enterobacter aerogenes KCTC 2190 uid68103	1171	1330	2411	4912	0.762	PMID: 22493190
Shigella sonnei 53G uid84383	1303	1586	2521	5410	0.759	https://www.ncbi.nlm.nih.gov/genome/?term=Shigella+sonnei+53G+uid84383
Treponema pallidum DAL 1 uid87065	256	404	396	1056	0.758	PMID: 23449808
Enterobacter cloacae SCF1 uid59969	1067	1249	2083	4399	0.757	PMC3236048
Moraxella catarrhalis BBH18 uid48809	460	452	974	1886	0.756	PMID: 20453089
Capnocytophaga canimorsus Cc5 uid70727	590	607	1207	2404	0.755	https://doi.org/10.1371/journal.ppat.1000164
Shigella flexneri 2,002,017 uid159233	1160	1239	2304	4703	0.753	PMID: 19955273
Nocardia brasiliensis ATCC 700,358 uid86913	2081	3731	2602	8414	0.753	PMC3347167
Salmonella typhimurium DT104 uid223287	1159	1280	2153	4592	0.748	PMID: 9752592

Where, Class 1: Non-pathogenic proteins; Class 2: Antibiotic resistance and toxic proteins and Class 3: Secretory and capsular proteins

**Table 8:** MP4 performance on the non-pathogenic protein

Strains	Class 1	Class 2	Class 3	Total sequences	Pathogenicity index
Thermotoga maritima MSB8 uid57723	1086	342	430	1858	0.416
Aquifex aeolicus VF5 uid57765	875	250	401	1526	0.427
Mycoplasma hyopneumoniae 7448 uid58039	291	95	271	657	0.557
Bacillus coagulans 2 6 uid68053	1273	639	1059	2971	0.572
Bacillus halodurans C 125 uid57791	1686	948	1418	4052	0.584
Thermoanaerobacterium thermosaccharolyticum DSM 571 uid51639	1017	752	832	2601	0.609
Bacillus licheniformis DSM 13 ATCC 14,580 uid58199	1608	949	1614	4171	0.614
Corynebacterium urealyticum DSM 7111 uid188688	744	522	669	1935	0.616
Lactobacillus fermentum CECT 5716 uid162003	403	217	431	1051	0.617
Staphylococcus carnosus TM300 uid59401	927	559	975	2461	0.623
Lactobacillus delbrueckii bulgaricus ATCC 11842 uid58647	573	255	701	1529	0.625
Listeria welshimeri serovar 6b SLCC5334 uid61605	1026	601	1147	2774	0.63
Listeria innocua Clip11262 uid61567	1113	649	1281	3043	0.634
Bacillus pumilus SAFR 032 uid59017	1341	754	1584	3679	0.635
Listeria ivanovii PAM 55 uid73473	960	549	1141	2650	0.638
Lactococcus garvieae ATCC 49156 uid73413	690	359	898	1947	0.646
Bacillus subtilis BSP1 uid184010	1362	846	1639	3847	0.646
Streptococcus parauberis KCTC 11537 uid67355	640	358	870	1868	0.657
Mycoplasma hyorhinis GDL 1 uid87003	219	135	293	647	0.662
Lactococcus lactis cremoris UC509 9 uid179384	701	414	994	2109	0.668
Lactococcus lactis IO 1 uid192185	739	440	1045	2224	0.668
Bacillus thuringiensis Al Hakam uid58795	1585	1333	1880	4798	0.67
Corynebacterium argentoratense DSM 44202 uid217419	614	506	755	1875	0.673
Pseudomonas putida S16 uid68747	1683	1388	2147	5218	0.677
Tetragenococcus halophilus uid74441	823	542	1190	2555	0.678

Where, Class 1: Non-pathogenic proteins; Class 2: Antibiotic resistance and toxic proteins and Class 3: Secretory and capsular proteins

**Table 9:** Class wise predictions obtained by MP4 on healthy and CRC affected individual's metagenomic data

Samples	Number of sequences	Percentage of Class 1 predictions	Percentage of Class 2 predictions	Percentage of Class 3 predictions	Pathogenicity index
Healthy sample	9569	29.33	25.47	45.17	0.70
CRC sample	9022	17.85	36.53	45.61	0.82

Where, Class 1: Non-pathogenic Proteins; Class 2: Antibiotic Resistance and Toxic proteins and Class 3: Secretory and capsular proteins

**Table 10:** Pathogenicity indices of various datasets based on prediction by MP4

Sequence datasets	Total sequences	Class 1	Class 2	Class 3	Pathogenicity index
VirulenPred dataset	40	0	10	30	1
*Shigella group1*	18	1	0	17	0.94
*Shigella group2*	19	5	6	8	0.74
*Shigella group3*	1	0	0	1	1

Where, Class 1: Non-pathogenic Proteins; Class 2: Antibiotic Resistance and Toxic proteins and Class 3: Secretory and capsular proteins

Using the CRC and healthy metagenomic samples, 9,022 and 9,569 sequences respectively were used as input for MP4 to obtain proportion of different classes of pathogenic proteins in the datasets. MP4 predicted a higher proportion of Class 1 proteins (non-pathogenic proteins) in healthy sample as compared to CRC sample. Similarly, the proportion of Class 2 proteins (antibiotic resistance proteins and toxins) was higher in CRC sample compared to the healthy sample. The proportion of Class 3 proteins (associated secretory system and capsular proteins) was found similar in the CRC and healthy samples. This was reasonably expected since the secretory systems and other associated structural proteins could be present in both pathogenic or non-pathogenic bacteria, however in case of pathogenic bacteria they are associated with host pathogenesis, and in non-pathogenic bacteria they serve as important features for survival in various environments. Overall, MP4 predicted higher pathogenicity index for CRC sample (0.82) as compared to healthy sample (0.71) (Table 9).

### Performance comparison between MP4 and MP3 using VirulentPred and MP3 datasets

The performance of MP4 was compared with MP3 and VirulentPred on the dataset obtained from VirulentPred. The MP3 and VirulentPred tools provided an accuracy of 90% and 85%, respectively, whereas MP4 provided an accuracy of 100%. on the same dataset (Table 10).

Another validation was performed using the dataset of pathogenic proteins obtained from MP3 [3]. For the proteins present on virulence plasmid of *Shigella group I* (translocated proteins), 17 out of 18 proteins were predicted to be pathogenic by both MP3 and MP4. Out of these 17, MP4 predicted all proteins except for *IpgB2* to be in the category of secretory and capsular proteins (Class 3) which is supported by the fact that virulent plasmid of *Shigella* group I are translocated proteins. In another case, *OspD3* protein was predicted to be non-pathogenic by MP3, however, MP4 predicted *OspD3* to be pathogenic (Class 3). This result was also supported by the BLAST analysis and the literature studies showing that it belongs to type III secretion system and can cause inflammation in the epithelial cells [37]. In case of *Shigella group II* (non-translocated proteins), 16 out of 20 known pathogenic proteins were predicted to be pathogenic by MP4, whereas MP3 predicted 12 out of the 20 proteins as pathogenic proteins. The *Shigella group III* consisted of *IpaJ* sequence, which was also predicted to be pathogenic by both MP4 and MP3 (Table 9, and Additional file 11: Table S7).

Thus, MP4 provided higher accuracy than MP3 and VirulentPred on the MP3 and VirulentPred datasets. Further, MP3 and VirulentPred could only classify the proteins as pathogenic or non-pathogenic, whereas MP4 classified the input proteins into pathogenic or non-pathogenic, and also provided a functional annotation for the classified proteins.

### Performance of MP4 on *Mycobacterium tuberculosis* NITR203 protein dataset.

Out of 41 proteins used in this dataset, both MP4 and MP3 were able to predict all these proteins to be pathogenic. While MP3 classified these proteins as pathogenic, MP4 was able to sub-categorize these proteins into either Class 2 or Class 3 pathogenic proteins. Lipoprotein LpqH anchored on cell membrane of *Mycobacterium tuberculosis* [38] was predicted to be pathogenic by MP3 and was correctly sub-categorized as Class 3 protein by MP4. Zinc metallopeptidase was another known pathogenic protein [39] which was correctly predicted to be pathogenic by MP3 but MP4 was able to classify it as Class 2 pathogenic protein. Moreover, MP4 was able to predict and classify many hypothetical proteins such as hypothetical protein MT2286, hypothetical protein MT2731 and hypothetical protein J112_13775 as Class 2 proteins and hypothetical proteins MRA_2260, J112_12965 and FJ05194_3111 as Class 3 pathogenic protein (Additional file 12: Table S8).

### Development of MP4 web server

**Fig. 1:**
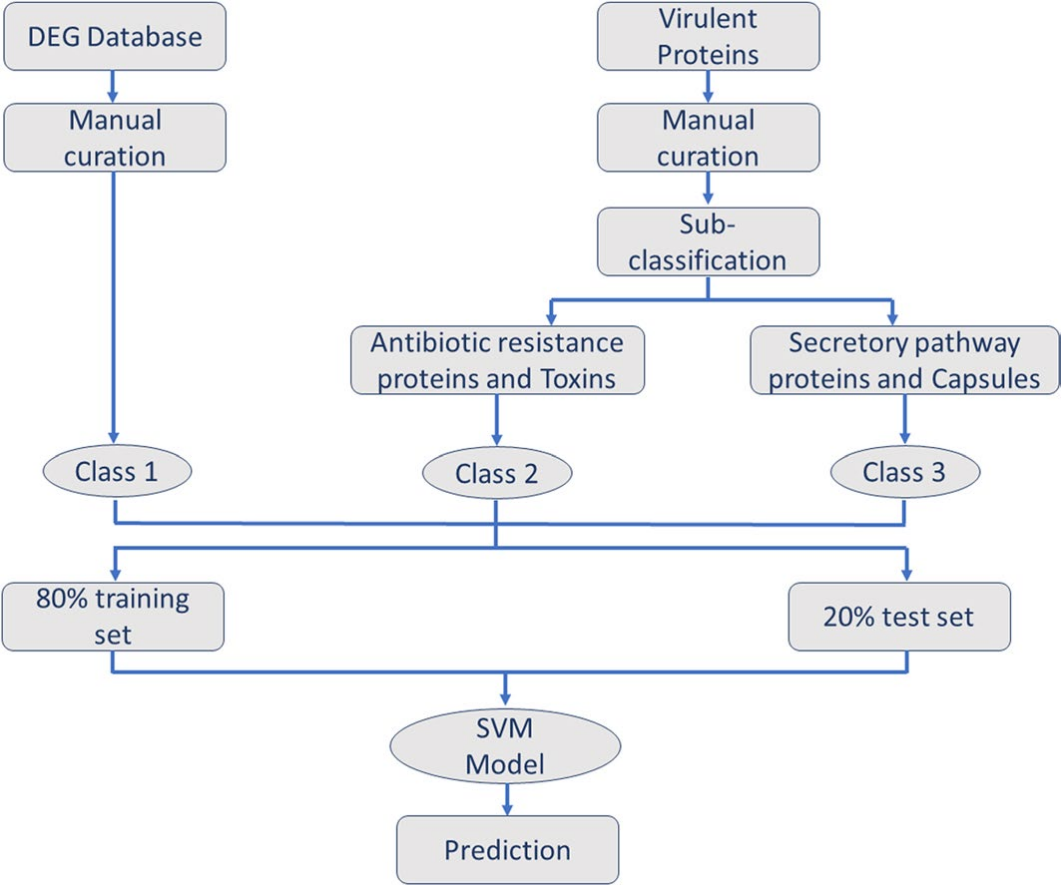
The steps involved in the construction of MP4 classification models for the prediction of pathogenic proteins

The steps involved in the development of a web interface for MP4 are shown in Fig. 1. Using the final classification models developed using the aforementioned processes, a user can predict the category of pathogenic protein. The prediction modules enable the users to input the information by pasting the sequences in FASTA format or by uploading the FASTA file. The query is analysed through the background model and the prediction is displayed on the Results page. The results can also be downloaded using the download link provided on the Results page. The web-server can be accessed at http://metagenomics.iiserb.ac.in/mp4/.

## Discussion

Functional annotation of pathogenic proteins requires tedious experimental procedures and validation which is a time-consuming and challenging task. In this case, homology-based approaches like BLAST can be used to assign functions to unknown proteins, however, these are majorly limited due to the availability of information in reference protein databases [14]. In this scenario, the machine learning based approaches provide valuable alternatives since multiple features from any biological input can be exploited to train the ML models etc. and thus can be used to construct efficient and reliable classifiers.

MP4, an SVM-based tool, developed in this study can help the users to predict pathogenic proteins and sub-classify the proteins based on their role in the process of pathogenesis. The tool can make reliable and accurate functional annotation of pathogenic proteins with comparatively higher sensitivity and specificity. The evaluation of performance on real dataset-1 and real dataset-2, on the independent metagenomic dataset, bacterial pathogenic and non-pathogenic datasets, and comparison of its performance with publicly available tools such as MP3 and VirulentPred attests to the accuracy and reliability of this tool. Additionally, while BLAST failed to provide annotations to hypothetical mycobacterial proteins, MP4 was able to identify and annotate such proteins and classify them into their respective class based on their function. Therefore, to the best of our knowledge, MP4 is currently the only available machine learning based tool that can predict and classify pathogenic proteins based on their function in any genomic or metagenomic dataset, and thus a wide usage of tool is anticipated.

## Supplementary Information

**Additional file 1**.** Table S1**: The complete list of variables with the mean decrease in accuracy values.

**Additional file 2**.** Table S2**: The different parameters used for the optimisation of the SVM based classifier.

**Additional file 3**.** Fig. S1**: Mean decrease in accuracy of top 30 features selected through random forest algorithm.

**Additional file 4**.** Table S3**: Performances of random forest-based models at top features and different mtry values.

**Additional file 5**.** Fig. S2a**: Optimization of random forest at various mtry and ntree values using dipeptide frequency and pepstats features as inputs, (a) performance using top 5% features, (b) performance using top 10% features, (c) performance using top 15% features, (d) performance using top 20% features.

**Additional file 6**.** Fig. S2b**: Optimization of random forest at various mtry and ntree values using dipeptide frequency and pepstats features as inputs, (a) performance using top 30% features (b) performance using top 50% features, (c) performance using top 70% features and (d) performance using top 90% features.

**Additional file 7**. List of important features obtained by VarImp function in SVM.

**Additional file 8. Table S4**: Blind set performance of best RF-based model.

**Additional file 9**.** Table S5**: Performance of MP4, MP3 and VirulentPred on real dataset-1.

**Additional file 10**.** Table S6**: Performance of MP4, MP3 and VirulentPred on real dataset-2.

**Additional file 11**.** Table S7**: Comparison results between MP4 and MP3 on *Shigella flexineri* virulence plasmid sequences.

**Additional file 12**.** Table S8**: Performance of MP4 on *Mycobacterium tuberculosis *NITR203 proteins.

## Data Availability

All the sequences used for training the SVM model are available on the MP4 homepage: http://metagenomics.iiserb.ac.in/mp4.

## Abbreviations

ML: Machine learning
SVM: Support vector machine
kNN: K-nearest neighbours
SVM-HMM: Support vector machine hidden Markov Model
PART: Partial decision tree algorithm
IBk: Instance based learner
RF: Random forest
MCC: Mathew’s correlation coefficient
OOB error: Out of box error
