# Supplementary material for: MP4: a machine learning based classification tool for prediction and functional annotation of pathogenic proteins from metagenomic and genomic datasets
Source: BMC Bioinformatics. 2022 Nov 28;23:507. doi: 10.1186/s12859-022-05061-7 (PMC9703692; doi:10.1186/s12859-022-05061-7)
Supplement: Supplementary file 1 — Additional file 1. Table S1: The complete list of variables with the mean decrease in accuracy values. [file 12859_2022_5061_MOESM1_ESM.docx]

Table S1: The complete list of variables with the mean decrease in accuracy values

| **Features** | **MDA** |
| --- | --- |
| Chd1 | 8.948767753 |
| S1 | 8.877688126 |
| A280 | 8.519568804 |
| R1 | 8.446175343 |
| W1 | 8.081700302 |
| CB1 | 8.006388671 |
| Ac1 | 7.318717634 |
| E1 | 6.886646952 |
| Ba1 | 6.690937249 |
| N1 | 6.358831172 |
| Ar1 | 6.22885456 |
| Q1 | 6.013779838 |
| K1 | 5.716653499 |
| V1 | 5.585883392 |
| I1 | 5.564985724 |
| Np1 | 5.524331411 |
| SS | 5.183640883 |
| L1 | 5.138186676 |
| Po1 | 5.124508487 |
| G1 | 5.10763176 |
| Ti1 | 4.96479778 |
| Ch1 | 4.894260962 |
| H1 | 4.847276687 |
| GR | 4.794467956 |
| Sm1 | 4.729619018 |
| Al1 | 4.703069886 |
| Y1 | 4.702998558 |
| RR | 4.661245539 |
| IG | 4.609110008 |
| T1 | 4.596278776 |
| IEP1 | 4.533076791 |
| C1 | 4.421438881 |
| LS | 4.332706867 |
| WE | 4.208993335 |
| VW | 4.099224081 |
| ER | 4.048948034 |
| ARW1 | 4.039630213 |
| VE | 4.024349471 |
| NN | 3.947881159 |
| VR | 3.943907286 |
| FG | 3.891851039 |
| WP | 3.876182853 |
| AW | 3.819603521 |
| EA | 3.812874859 |
| A1 | 3.81205851 |
| F1 | 3.702977867 |
| LI | 3.657708134 |
| VG | 3.596704857 |
| TT | 3.576368005 |
| RL | 3.52059062 |
| LF | 3.514528615 |
| RV | 3.505642769 |
| IE | 3.493137948 |
| GC | 3.485296973 |
| AE | 3.443787434 |
| QE | 3.433487878 |
| P1 | 3.432924671 |
| RS | 3.428824287 |
| GG | 3.331284136 |
| GP | 3.32847288 |
| D1 | 3.318034145 |
| LL | 3.315431137 |
| DR | 3.312057463 |
| EV | 3.308088809 |
| YG | 3.301376762 |
| PW | 3.295925217 |
| HT | 3.282470192 |
| SA | 3.276735029 |
| PE | 3.267312601 |
| RW | 3.222256529 |
| RT | 3.204253 |
| WL | 3.177456864 |
| M1 | 3.143532796 |
| NL | 3.132661133 |
| IR | 3.120765708 |
| CS | 3.096846218 |
| CR | 3.075849101 |
| SG | 3.070420888 |
| TS | 3.045148399 |
| DW | 3.031699004 |
| TF | 3.031674956 |
| AS | 3.023866049 |
| IW | 3.02023942 |
| FE | 3.01839896 |
| LR | 3.002194534 |
| QG | 2.983718443 |
| AV | 2.97511984 |
| AQ | 2.971364838 |
| LE | 2.968596173 |
| SI | 2.958524342 |
| YV | 2.957728622 |
| AD | 2.949514734 |
| TL | 2.935839079 |
| RF | 2.934693112 |
| LG | 2.926701862 |
| RQ | 2.924098664 |
| RP | 2.907249497 |
| AC | 2.899280735 |
| GA | 2.894220427 |
| CQ | 2.888565149 |
| NS | 2.88025068 |
| KY | 2.873136805 |
| KN | 2.85818453 |
| EI | 2.852156863 |
| EW | 2.852043865 |
| GH | 2.84968698 |
| ST | 2.838683873 |
| EE | 2.831513507 |
| KA | 2.831412617 |
| SL | 2.830872568 |
| YN | 2.816527769 |
| FS | 2.816229731 |
| CC | 2.815796507 |
| WR | 2.80127717 |
| KL | 2.79704272 |
| HI | 2.79560833 |
| WK | 2.789617557 |
| DA | 2.781587081 |
| NG | 2.778463377 |
| LQ | 2.773732934 |
| WF | 2.773006415 |
| YS | 2.771299265 |
| RA | 2.766193526 |
| DE | 2.743653159 |
| AH | 2.718501924 |
| WG | 2.710809882 |
| RC | 2.700739783 |
| TG | 2.699793831 |
| NP | 2.697074289 |
| GQ | 2.688428118 |
| RH | 2.665931148 |
| GN | 2.66470974 |
| NI | 2.654860053 |
| QI | 2.632188255 |
| DL | 2.626448685 |
| FL | 2.625200252 |
| RI | 2.617260609 |
| II | 2.616376453 |
| LM | 2.610826777 |
| AR | 2.603961331 |
| AG | 2.584750856 |
| IL | 2.579164319 |
| TN | 2.570114476 |
| DH | 2.568494135 |
| WQ | 2.568319671 |
| HL | 2.566200223 |
| EL | 2.566046251 |
| LK | 2.556421054 |
| LT | 2.554669599 |
| GD | 2.553714136 |
| RK | 2.547792836 |
| PP | 2.547662811 |
| DK | 2.537000945 |
| HG | 2.515371201 |
| FR | 2.515193687 |
| NA. | 2.511717172 |
| AK | 2.508665456 |
| EQ | 2.507942833 |
| EG | 2.504631421 |
| PH | 2.501506466 |
| GW | 2.489792413 |
| QT | 2.477084497 |
| CL | 2.465772354 |
| RD | 2.458859412 |
| HP | 2.456891073 |
| LA | 2.45685844 |
| YF | 2.455440468 |
| PG | 2.449988096 |
| YP | 2.447432377 |
| WS | 2.446014949 |
| GT | 2.443834758 |
| VN | 2.421866122 |
| PQ | 2.420912372 |
| SP | 2.420430729 |
| DF | 2.402877337 |
| CE | 2.40098746 |
| SR | 2.398229439 |
| SW | 2.397463373 |
| WM | 2.395744321 |
| SE | 2.395314362 |
| DV | 2.391533923 |
| ED | 2.386267316 |
| VF | 2.380069856 |
| LH | 2.378633816 |
| TM | 2.375218786 |
| SN | 2.368432574 |
| WN | 2.357423219 |
| QR | 2.356761621 |
| YA | 2.351028453 |
| VI | 2.34771212 |
| NT | 2.338795191 |
| HF | 2.337673795 |
| TD | 2.327230513 |
| QL | 2.324266524 |
| AF | 2.323279017 |
| HS | 2.323126922 |
| YW | 2.320739205 |
| NK | 2.320265147 |
| QH | 2.314882116 |
| SQ | 2.314483419 |
| RG | 2.313254443 |
| MS | 2.308676895 |
| QN | 2.307575403 |
| KH | 2.307396228 |
| LP | 2.305644674 |
| HY | 2.304972979 |
| KQ | 2.302803168 |
| TV | 2.298296394 |
| LN | 2.297909013 |
| TP | 2.292561491 |
| MA | 2.290536626 |
| RY | 2.289779224 |
| PR | 2.286611487 |
| FQ | 2.279998151 |
| KD | 2.270560887 |
| QQ | 2.269778973 |
| FY | 2.266418377 |
| LY | 2.257848956 |
| VY | 2.256234151 |
| HQ | 2.253240094 |
| TW | 2.249441565 |
| SH | 2.247528855 |
| GS | 2.235574718 |
| RE | 2.235295129 |
| DP | 2.231217846 |
| HR | 2.229869395 |
| ML | 2.22246264 |
| GY | 2.220390761 |
| DD | 2.219757126 |
| FK | 2.212972045 |
| KG | 2.21288408 |
| VP | 2.209199486 |
| QS | 2.203224155 |
| KV | 2.202146039 |
| VL | 2.198996146 |
| QA | 2.193339633 |
| EK | 2.192055676 |
| TA | 2.191780113 |
| EP | 2.191671161 |
| YL | 2.191493962 |
| FN | 2.180651619 |
| WV | 2.174393041 |
| LC | 2.167840852 |
| AP | 2.166088032 |
| FD | 2.16427179 |
| WY | 2.161093794 |
| YI | 2.157115345 |
| GK | 2.150378879 |
| KR | 2.150030565 |
| NQ | 2.148149098 |
| TC | 2.130585051 |
| GF | 2.129720411 |
| LW | 2.127092148 |
| IY | 2.114679428 |
| KK | 2.101603923 |
| LV | 2.085126469 |
| HH | 2.075118649 |
| FI | 2.074365136 |
| FW | 2.065160383 |
| HD | 2.064025227 |
| HC | 2.037377823 |
| VD | 2.027161948 |
| EM | 2.026154146 |
| EH | 2.018309868 |
| GL | 2.018013969 |
| YT | 2.016227316 |
| WT | 2.012494902 |
| MP | 2.01118553 |
| PI | 2.001144039 |
| PT | 1.987541247 |
| NV | 1.982044295 |
| VM | 1.979993736 |
| MI | 1.960145357 |
| NH | 1.959005313 |
| VK | 1.956864084 |
| PA | 1.952141931 |
| ME | 1.950396998 |
| IP | 1.941721935 |
| WC | 1.93744609 |
| TY | 1.932245291 |
| FH | 1.930276765 |
| SD | 1.926173431 |
| DQ | 1.925339237 |
| EF | 1.925122482 |
| CV | 1.924904135 |
| IA | 1.920957244 |
| SY | 1.917619049 |
| IM | 1.907050621 |
| NE | 1.903966162 |
| QY | 1.900656101 |
| IT | 1.889284534 |
| MQ | 1.888087209 |
| KI | 1.886094434 |
| CG | 1.878618382 |
| IF | 1.876110888 |
| YQ | 1.870558719 |
| YH | 1.868434815 |
| CH | 1.867338781 |
| WA | 1.86460187 |
| VA | 1.861680693 |
| VV | 1.850563757 |
| SV | 1.848825567 |
| MW | 1.846723869 |
| MY | 1.845486845 |
| YY | 1.838648441 |
| PS | 1.829784308 |
| TQ | 1.8259958 |
| IK | 1.811739368 |
| IV | 1.799862593 |
| DI | 1.794580624 |
| IS | 1.792841239 |
| NF | 1.79174773 |
| QV | 1.784258899 |
| CY | 1.782599785 |
| HK | 1.773042283 |
| YD | 1.772236829 |
| QK | 1.758814986 |
| DT | 1.753537292 |
| TH | 1.751809978 |
| MF | 1.751142427 |
| DG | 1.749179621 |
| HM | 1.746689168 |
| NW | 1.745867983 |
| ID | 1.738978832 |
| FT | 1.7218101 |
| WW | 1.692796696 |
| QP | 1.685512107 |
| AY | 1.684666434 |
| YM | 1.681404994 |
| HA | 1.664941194 |
| RN | 1.660088349 |
| WD | 1.654764256 |
| MD | 1.65390679 |
| SK | 1.653806157 |
| IC | 1.646044303 |
| NC | 1.642796361 |
| HV | 1.639027945 |
| QW | 1.638688823 |
| FA | 1.630930419 |
| VT | 1.6194132 |
| HE | 1.614208714 |
| MC | 1.607530107 |
| KW | 1.596493562 |
| NY | 1.588272333 |
| QM | 1.582414581 |
| DN | 1.580785218 |
| MH | 1.5782735 |
| LD | 1.568979234 |
| IH | 1.562333351 |
| WI | 1.559204174 |
| PM | 1.54618921 |
| FP | 1.537618187 |
| SF | 1.535200008 |
| CF | 1.534970104 |
| ET | 1.528535855 |
| PD | 1.517083722 |
| MK | 1.51156887 |
| PL | 1.500246606 |
| KP | 1.49028577 |
| CN | 1.478334219 |
| PV | 1.465801774 |
| DC | 1.458522172 |
| FF | 1.456303397 |
| YE | 1.455001896 |
| PF | 1.443017287 |
| AL | 1.433691179 |
| FC | 1.432651852 |
| CD | 1.427680952 |
| KS | 1.425520571 |
| HW | 1.421401874 |
| GE | 1.415836666 |
| QD | 1.392051629 |
| MT | 1.384984311 |
| AM | 1.383181055 |
| AN | 1.381893986 |
| KF | 1.379767286 |
| EY | 1.371385228 |
| PC | 1.359680477 |
| MR | 1.359666446 |
| VH | 1.358827108 |
| FM | 1.353806551 |
| GI | 1.351957363 |
| PY | 1.350027124 |
| IQ | 1.341142477 |
| YK | 1.332246774 |
| AI | 1.323507835 |
| CP | 1.314237161 |
| GV | 1.308025247 |
| PN | 1.302454159 |
| TI | 1.294992375 |
| GM | 1.290564452 |
| MG | 1.289247932 |
| EN | 1.285022942 |
| VQ | 1.282279477 |
| HN | 1.270792809 |
| KC | 1.269327665 |
| ND | 1.268226888 |
| QF | 1.26208503 |
| AT | 1.261989583 |
| DS | 1.251190308 |
| CA | 1.243465808 |
| SC | 1.240230418 |
| CI | 1.207559979 |
| KT | 1.205435243 |
| QC | 1.205434826 |
| PK | 1.182766525 |
| YC | 1.171344145 |
| VC | 1.167894356 |
| MN | 1.164247507 |
| KE | 1.163782878 |
| IN | 1.143429631 |
| DY | 1.129772211 |
| WH | 1.12112321 |
| KM | 1.114293666 |
| MV | 1.088830768 |
| TR | 1.076356337 |
| TK | 1.073962145 |
| ES | 1.006228791 |
| CM | 0.987958997 |
| YR | 0.978395711 |
| DM | 0.920922575 |
| NM | 0.900102643 |
| FV | 0.896072101 |
| CW | 0.871148726 |
| SM | 0.857690235 |
| TE | 0.806836507 |
| NR | 0.742071301 |
| MM | 0.708905538 |
| VS | 0.671932739 |
| RM | 0.584073146 |
| CK | 0.501738236 |
| CT | 0.496899656 |
| EC | 0.446782571 |

Where MDA: mean decrease in accuracy
