# Supplementary material for: MP4: a machine learning based classification tool for prediction and functional annotation of pathogenic proteins from metagenomic and genomic datasets
Source: BMC Bioinformatics. 2022 Nov 28;23:507. doi: 10.1186/s12859-022-05061-7 (PMC9703692; doi:10.1186/s12859-022-05061-7)
Supplement: Supplementary file 2 — Additional file 2. Table S2: The different parameters used for the optimisation of the SVM based classifier. [file 12859_2022_5061_MOESM2_ESM.docx]

# Table S2: The different parameters used for the optimisation of the SVM based classifier.

| **Kernel** | **Cost Factor (C)** | **Coefficient**  **(coef0)** | **Gamma value** | **Degree** |
| --- | --- | --- | --- | --- |
| Linear | 1, 5, 10, 15, 23, 28, 33, 37, 35, 54, 45, 42, 49, 52, 59, 66, 69, 73, 76, 79, 84, 89, 93, 98 | - | - | - |
| Polynomial | 1, 5, 10, 15, 23, 28, 33, 37, 35, 54, 45, 42, 49, 52, 59, 66, 69, 73, 76, 79, 84, 89, 93, 98 | 1, 2, 3, 4, 5, 6, 7, 8, 9 | 10^(-3:3)^ | 2, 3, 4 |
| RBF | 1, 5, 10, 15, 23, 28, 33, 37, 35, 54, 45, 42, 49, 52, 59, 66, 69, 73, 76, 79, 84, 89, 93, 98 | - | 10^(-3:3)^ | - |

Where, RBF: Radial Basis Function
