# Supplementary figures and images for: MP4: a machine learning based classification tool for prediction and functional annotation of pathogenic proteins from metagenomic and genomic datasets
Source: BMC Bioinformatics. 2022 Nov 28;23:507. doi: 10.1186/s12859-022-05061-7 (PMC9703692; doi:10.1186/s12859-022-05061-7)

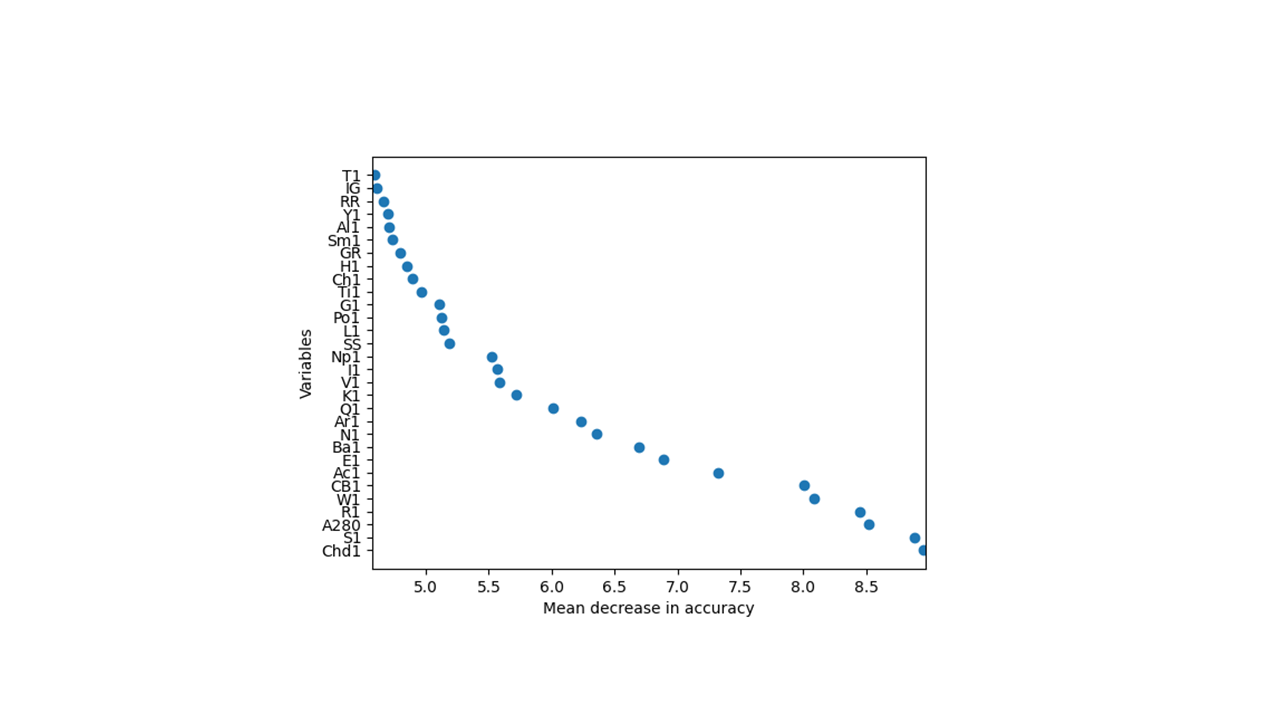

Supplement: Supplementary file 3 — Additional file 3. Fig. S1: Mean decrease in accuracy of top 30 features selected through random forest algorithm. [file 12859_2022_5061_MOESM3_ESM.tif]

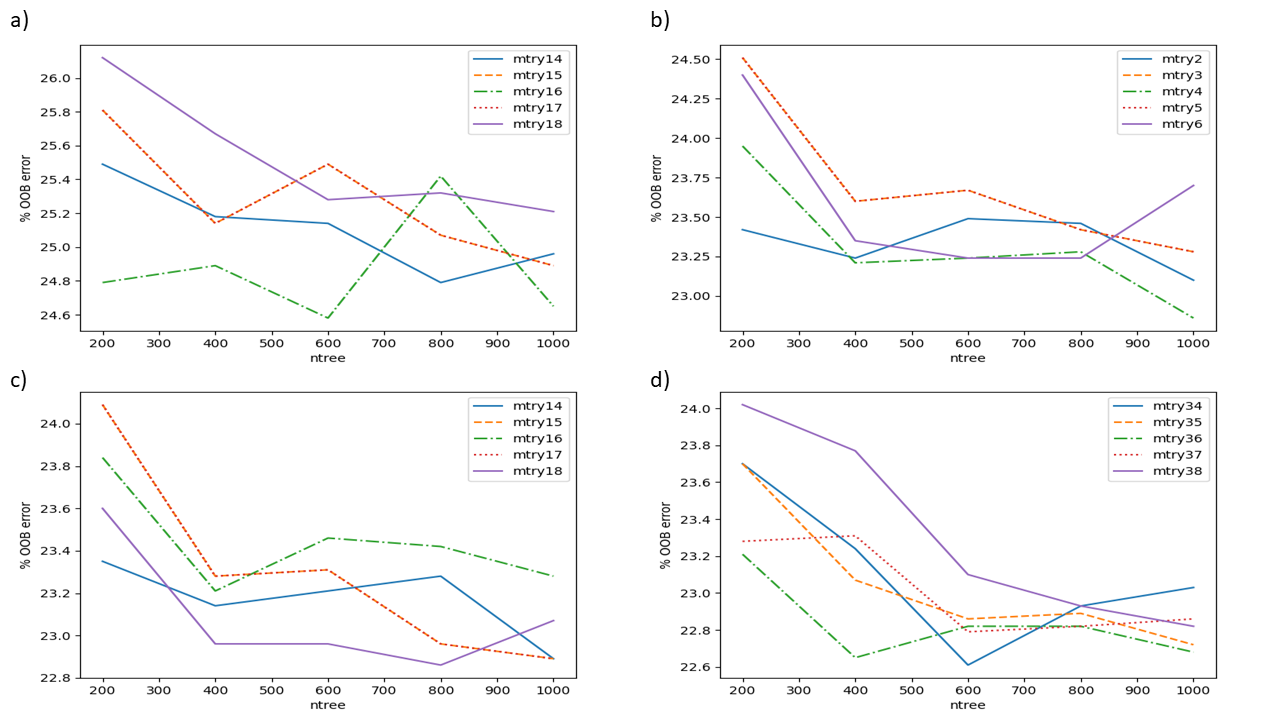

Supplement: Supplementary file 5 — Additional file 5. Fig. S2a: Optimization of random forest at various mtry and ntree values using dipeptide frequency and pepstats features as inputs, (a) performance using top 5% features, (b) performance using top 10% features, (c) performance using top 15% features, (d) performance using top 20% features. [file 12859_2022_5061_MOESM5_ESM.tif]

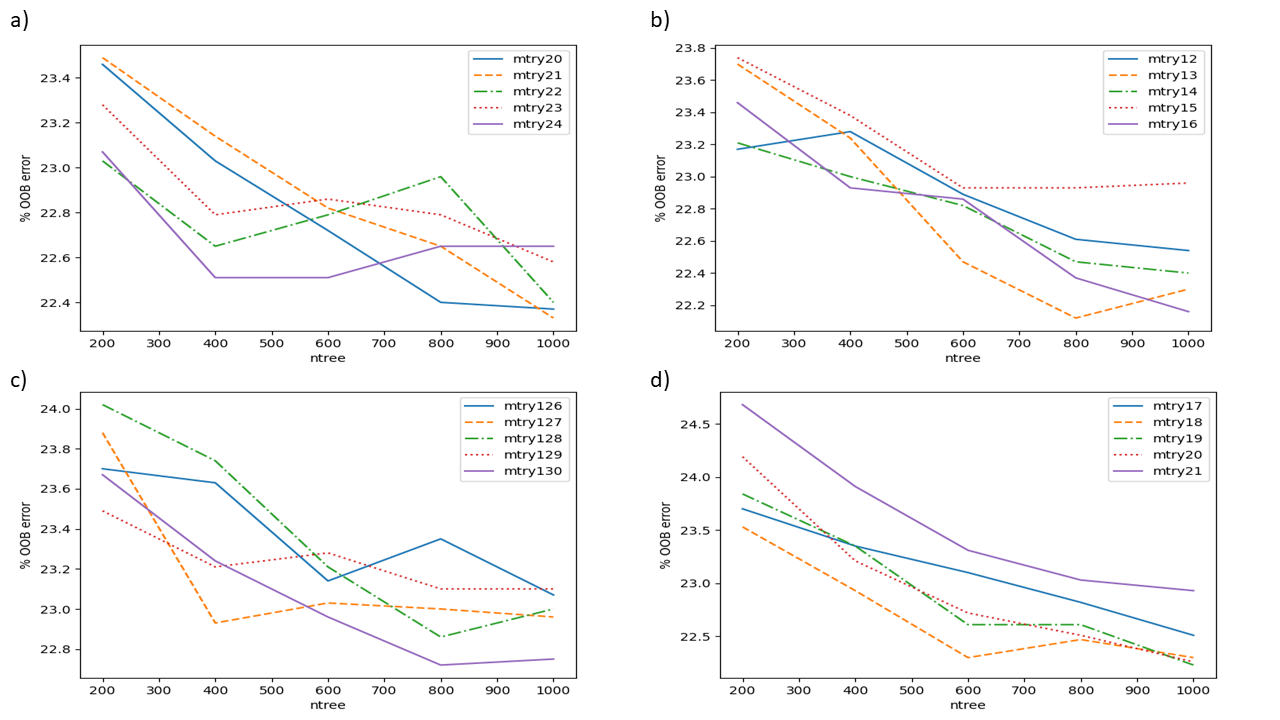

Supplement: Supplementary file 6 — Additional file 6. Fig. S2b: Optimization of random forest at various mtry and ntree values using dipeptide frequency and pepstats features as inputs, (a) performance using top 30% features (b) performance using top 50% features, (c) performance using top 70% features and (d) performance using top 90% features. [file 12859_2022_5061_MOESM6_ESM.tif]
