# Supplementary material for: MP4: a machine learning based classification tool for prediction and functional annotation of pathogenic proteins from metagenomic and genomic datasets
Source: BMC Bioinformatics. 2022 Nov 28;23:507. doi: 10.1186/s12859-022-05061-7 (PMC9703692; doi:10.1186/s12859-022-05061-7)
Supplement: Supplementary file 4 — Additional file 4. Table S3: Performances of random forest-based models at top features and different mtry values. [file 12859_2022_5061_MOESM4_ESM.doc]

| **mtry** | **ntree200** | **ntree400** | **ntree600** | **ntree800** | **ntree1000** | **Features (%)** |
| --- | --- | --- | --- | --- | --- | --- |
| 14 | 25.49 | 25.18 | 25.14 | 24.79 | 24.96 | Top5 |
| 15 | 25.81 | 25.14 | 25.49 | 25.07 | 24.89 |
| 16 | 24.79 | 24.89 | 24.58 | 25.42 | 24.65 |
| 17 | 25.81 | 25.14 | 25.49 | 25.07 | 24.89 |
| 18 | 26.12 | 25.67 | 25.28 | 25.32 | 25.21 |
| 2 | 23.42 | 23.24 | 23.49 | 23.46 | 23.1 | Top10 |
| 3 | 24.51 | 23.6 | 23.67 | 23.42 | 23.28 |
| 4 | 23.95 | 23.21 | 23.24 | 23.28 | 22.86 |
| 5 | 24.51 | 23.6 | 23.67 | 23.42 | 23.28 |
| 6 | 24.4 | 23.35 | 23.24 | 23.24 | 23.7 |
| 14 | 23.35 | 23.14 | 23.21 | 23.28 | 22.89 | Top15 |
| 15 | 24.09 | 23.28 | 23.31 | 22.96 | 22.89 |
| 16 | 23.84 | 23.21 | 23.46 | 23.42 | 23.28 |
| 17 | 24.09 | 23.28 | 23.31 | 22.96 | 22.89 |
| 18 | 23.6 | 22.96 | 22.96 | 22.86 | 23.07 |
| 34 | 23.7 | 23.24 | 22.61 | 22.93 | 23.03 | Top20 |
| 35 | 23.7 | 23.07 | 22.86 | 22.89 | 22.72 |
| 36 | 23.21 | 22.65 | 22.82 | 22.82 | 22.68 |
| 37 | 23.28 | 23.31 | 22.79 | 22.82 | 22.86 |
| 38 | 24.02 | 23.77 | 23.1 | 22.93 | 22.82 |
| 20 | 23.46 | 23.03 | 22.72 | 22.4 | 22.37 | Top30 |
| 21 | 23.49 | 23.14 | 22.82 | 22.65 | 22.33 |
| 22 | 23.03 | 22.65 | 22.79 | 22.96 | 22.4 |
| 23 | 23.28 | 22.79 | 22.86 | 22.79 | 22.58 |
| 24 | 23.07 | 22.51 | 22.51 | 22.65 | 22.65 |
| 12 | 23.17 | 23.28 | 22.89 | 22.61 | 22.54 | Top50 |
| 13 | 23.7 | 23.24 | 22.47 | 22.12 | 22.3 |
| 14 | 23.21 | 23 | 22.82 | 22.47 | 22.4 |
| 15 | 23.74 | 23.38 | 22.93 | 22.93 | 22.96 |
| 16 | 23.46 | 22.93 | 22.86 | 22.37 | 22.16 |
| 126 | 23.7 | 23.63 | 23.14 | 23.35 | 23.07 | Top70 |
| 127 | 23.88 | 22.93 | 23.03 | 23 | 22.96 |
| 128 | 24.02 | 23.74 | 23.21 | 22.86 | 23 |
| 129 | 23.49 | 23.21 | 23.28 | 23.1 | 23.1 |
| 130 | 23.67 | 23.24 | 22.96 | 22.72 | 22.75 |
| 17 | 23.7 | 23.35 | 23.1 | 22.82 | 22.51 | Top90 |
| 18 | 23.53 | 22.93 | 22.3 | 22.47 | 22.3 |
| 19 | 23.84 | 23.35 | 22.61 | 22.61 | 22.23 |
| 20 | 24.19 | 23.21 | 22.72 | 22.51 | 22.26 |
| 21 | 24.68 | 23.91 | 23.31 | 23.03 | 22.93 |

Table S3: Performances of random forest based models at top features and different mtry values

Where, model at top 50% features, mtry= 13 and ntree= 800, gave the best performance
