# Supplementary material for: MP4: a machine learning based classification tool for prediction and functional annotation of pathogenic proteins from metagenomic and genomic datasets
Source: BMC Bioinformatics. 2022 Nov 28;23:507. doi: 10.1186/s12859-022-05061-7 (PMC9703692; doi:10.1186/s12859-022-05061-7)
Supplement: Supplementary file 7 — Additional file 7. List of important features obtained by VarImp function in SVM. [file 12859_2022_5061_MOESM7_ESM.docx]

Text S1: List of important features obtained by VarImp function in SVM

A280,AA,Ac,AC,AD,AE,AF,AG,AH,AI,AK,Al,AL,AM,AN,AP,AQ,Ar,AR,ARW,AS,AT,AV,AW,AY,Ba,C1,CB,CC,CE,CF,CG,Ch,Chd,CL,CP,CQ,CR,CS,CV,CY,D1,DA,DC,DD,DE,DF,DG,DH,DI,DK,DL,DM,DN,DP,DQ,DR,DS,DV,DW,DY,E1,EA,EC,ED,EE,EF,EG,EH,EI,EK,EL,EM,EN,EP,EQ,ER,ES,ET,EV,EW,EY,F1,FA,FD,FE,FF,FG,FH,FI,FK,FL,FN,FP,FQ,FR,FS,FT,FV,FW,FY,G1,GA,GC,GD,GE,GF,GG,GH,GI,GK,GL,GN,GP,GQ,GR,GS,GV,GW,GY,H1,HA,HC,HE,HF,HG,HH,HI,HK,HL,HM,HN,HP,HR,HS,HT,HV,HW,HY,I1,IA,IC,ID,IE,IEP,IF,IG,IH,II,IK,IL,IN,IP,IQ,IR,IS,IV,IW,IY,K1,KA,KD,KE,KF,KG,KI,KK,KL,KM,KN,KP,KQ,KR,KS,KT,KV,KW,KY,L1,LC,LD,LE,LF,LG,LH,LI,LK,LL,LM,LN,LP,LQ,LR,LS,LT,LV,LW,LY,M1,MA,MD,ME,MG,MH,MI,MK,ML,MP,MQ,MR,MS,MV,MW,MY,N1,NA,NC,ND,NE,NF,NG,NI,NK,NL,NM,NN,NP,Np1,NQ,NS,NT,NW,P1,PA,PC,PD,PE,PF,PG,PH,PI,PK,PM,Po,PP,PQ,PR,PS,PT,PV,PW,PY,Q1,QA,QD,QE,QF,QI,QK,QL,QM,QN,QP,QQ,QR,QS,QT,QV,QW,QY,R1,RA,RC,RD,RE,RF,RG,RH,RI,RK,RL,RM,RN,RP,RQ,RR,RS,RT,RV,RW,RY,S1,SA,SD,SE,SF,SG,SH,SI,SK,SL,Sm,SN,SP,SQ,SR,SS,ST,SV,SW,SY,T1,TA,TD,TE,TF,TG,TH,Ti,TI,TK,TL,TM,TN,TP,TQ,TR,TS,TT,TV,TW,TY,V1,VA,VC,VD,VE,VF,VG,VH,VI,VK,VL,VM,VN,VP,VQ,VR,VS,VV,VW,VY,W1,WA,WD,WE,WF,WG,WI,WK,WL,WN,WP,WQ,WR,WS,WT,WV,WW,Y1,YA,YD,YE,YF,YG,YH,YK,YM,YN,YP,YQ,YR,YS,YT,YV,YW

Where, A280 is the best performing variable and YW is the least performing variable
