# Supplementary material for: MP4: a machine learning based classification tool for prediction and functional annotation of pathogenic proteins from metagenomic and genomic datasets
Source: BMC Bioinformatics. 2022 Nov 28;23:507. doi: 10.1186/s12859-022-05061-7 (PMC9703692; doi:10.1186/s12859-022-05061-7)
Supplement: Supplementary file 8 — Additional file 8. Table S4: Blind set performance of best RF-based model. [file 12859_2022_5061_MOESM8_ESM.docx]

Table S4: Blind set performance of best RF-based model

| Accuracy | 78.48 | | |
| --- | --- | --- | --- |
|  | **Class 1** | **Class 2** | **Class 3** |
| Sensitivity | 0.7395 | 0.6717 | 0.8926 |
| Specificity | 0.9415 | 0.9571 | 0.7530 |
| Balanced Accuracy | 0.8405 | 0.8144 | 0.8228 |

Where, **Class1**: Non-pathogenic Proteins; **Class 2**: Antibiotic resistant proteins and Toxin and **Class 3**: Secretory and capsular proteins
