# Supplementary material for: MP4: a machine learning based classification tool for prediction and functional annotation of pathogenic proteins from metagenomic and genomic datasets
Source: BMC Bioinformatics. 2022 Nov 28;23:507. doi: 10.1186/s12859-022-05061-7 (PMC9703692; doi:10.1186/s12859-022-05061-7)
Supplement: Supplementary file 11 — Additional file 11. Table S7: Comparison results between MP4 and MP3 on Shigella flexineri virulence plasmid sequences. [file 12859_2022_5061_MOESM11_ESM.docx]

Table S7: Comparison results between MP4 and MP3 on *Shigella flexineri* virulence plasmid sequences

| **Group** | **Protein** | **Function** | **Inhibition** | **MP3 prediction** | **MP4 Prediction** |
| --- | --- | --- | --- | --- | --- |
| Translocated | icsA | N-WASP recruiting protein |  | Pathogenic | Class 3 |
|  | icsB | Inhibits autophagy | Complete | Pathogenic | Class 3 |
|  | ipaA | Vinculin binding protein |  | Pathogenic | Class 3 |
|  | IpaH1.4 | Putative E3 ubiquitin ligase |  | Pathogenic | Class 3 |
|  | IpaH4.5 | Putative E3 ubiquitin ligase |  | Pathogenic | Class 3 |
|  | IpaH7.8 | Putative E3 ubiquitin ligase |  | Pathogenic | Class 3 |
|  | IpaH9.8 | E3 ubiquitin ligase |  | Pathogenic | Class 3 |
|  | IpgB1 | G-protein mimic | Intermediate | Pathogenic | Class 3 |
|  | IpgB2 | G-protein mimic | Complete | Pathogenic | Class1 |
|  | IpgD | Inositol phosphate phosphatase | Complete | Pathogenic | Class 3 |
|  | OspB | Unknown | Weak | Pathogenic | Class 3 |
|  | OspC1 | Unknown | Intermediate | Pathogenic | Class 3 |
|  | OspD1 | Unknown |  | Pathogenic | Class 3 |
|  | OspD2 | Unknown |  | Pathogenic | Class 3 |
|  | OspD3 | Unknown | Intermediate | Non-pathogenic | Class 3 |
|  | OspE2 | CO-localizes with focal contacts |  | Pathogenic | Class 3 |
|  | OspF | MAPK phosphothreonine | Intermediate | Pathogenic | Class 3 |
|  | VirA | Microtubule-severing activity | Complete | Pathogenic | Class 3 |
| Non-translocated | acp | Periplasmic acid phosphatase |  | Non-pathogenic | Class 3 |
|  | ccdA | Antitoxin-plasmid segregation |  | Non-pathogenic | Class 3 |
|  | finO | Transfer of plasmids |  | Non-pathogenic | Class 1 |
|  | ipgA | TTSS chaperone |  | Pathogenic | Class 2 |
|  | ipgC | TTSS chaperone |  | Pathogenic | Class 3 |
|  | ipgE | TTSS chaperone |  | Pathogenic | Class 2 |
|  | ipgF | TTSS chaperone |  | Non-pathogenic | Class 3 |
|  | msbB2 | Lipid A modification |  | Non-pathogenic | Class 3 |
|  | mvpA | Antitoxin-plasmid segregation |  | Non-pathogenic | Class 1 |
|  | mvpT | Toxin- plasmid segregation | Complete | Non-pathogenic | Class 2 |
|  | parA | Plasmid segregation | Intermediate | Non-pathogenic | Class 3 |
|  | parB | Plasmid segregation |  | Non-pathogenic | Class 1 |
|  | PhoN2 | Periplasmic acid phosphatase |  | Non-pathogenic | Class 3 |
|  | stbA | Plasmid segregation |  | Non-pathogenic | Class 2 |
|  | stbB | Plasmid segregation |  | Pathogenic | Class 3 |
|  | traX | Transfer of plasmids |  | Pathogenic | Class 2 |
|  | ushA | Periplasmic UDP-sugar hydrolase |  | Non-pathogenic | Class 2 |
|  | virB | Transcriptional activator |  | Non-pathogenic | Class 3 |
|  | virF | Transcriptional activator |  | Pathogenic | Class 2 |
| Group 3 | IpaJ | Unknown | Complete | Pathogenic | Class 3 |

Where, **Class 1:** Non-pathogenic Proteins (Non-pathogenic); **Class 2:** Antibiotic Resistance proteins and Toxins; **Class 3:** Secretory and Capsular Proteins
